# Inhibition of gut digestive proteases by cyanobacterial diets decreases infection in a *Daphnia* host–parasite system

**DOI:** 10.1002/ece3.11340

**Published:** 2024-04-19

**Authors:** Kristel F. Sánchez, Eric von Elert, Kira Monell, Siobhan Calhoun, Aniqa Maisha, Paige McCreadie, Meghan A. Duffy

**Affiliations:** ^1^ Department of Ecology and Evolutionary Biology University of Michigan Ann Arbor Michigan USA; ^2^ Department of Aquatic Chemical Ecology University of Cologne Cologne Germany

**Keywords:** ascospore, co‐opt, cyanobacteria, cyanopeptolins, *Microcystis aeruginosa*, pathogen, protease inhibitors

## Abstract

Secondary metabolites produced by primary producers have a wide range of functions as well as indirect effects outside the scope of their direct target. Research suggests that protease inhibitors produced by cyanobacteria influence grazing by herbivores and may also protect against parasites of cyanobacteria. In this study, we asked whether those same protease inhibitors produced by cyanobacteria could also influence the interactions of herbivores with their parasites. We used the *Daphnia*‐*Metschnikowia* zooplankton host‐fungal parasite system to address this question because it is well documented that cyanobacteria protease inhibitors suppress trypsin and chymotrypsin in the gut of *Daphnia*, and because it is known that *Metschnikowia* infects via the gut. We tested the hypothesis that *Daphnia* gut proteases are necessary for *Metschnikowia* spores to be released from their asci. We then also tested whether diets that decrease trypsin and chymotrypsin activity in the guts of *Daphnia* lead to lower levels of infection. Our results show that chymotrypsin promotes the release of the fungal spores from their asci. Moreover, a diet that strongly inhibited chymotrypsin activity in *Daphnia* decreased infection levels, particularly in the most susceptible *Daphnia* clones. Our results support the growing literature that cyanobacterial diets can be beneficial to zooplankton hosts when challenged by parasites and uncover a mechanism that contributes to the protective effect of cyanobacterial diets. Specifically, we demonstrate that host chymotrypsin enzymes promote the dehiscence of *Metschnikowia* spores; when cyanobacteria inhibit the activity of chymotrypsin in hosts, this most likely traps the spore inside the ascus, preventing the parasite from puncturing the gut and beginning the infection process. This study illustrates how secondary metabolites of phytoplankton can protect herbivores against their own enemies.

## Graphical abstract

Cyanobacteria peptides can inhibit gut enzymes in zooplankton hosts. Here, we present evidence that inhibition of these enzymes by cyanobacteria leads to lower infection prevalence in hosts that are susceptible to a fungal parasite.

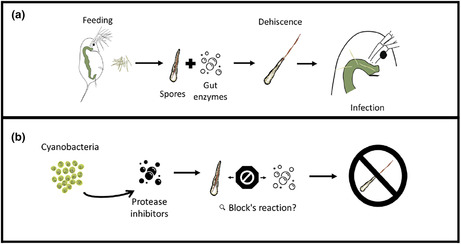

## 1 INTRODUCTION

Secondary metabolites are ubiquitous in nature, with impacts that span scales (Hunter, 2016). At smaller scales, secondary metabolites modulate interactions between organisms, such as when a primary producer produces fouling chemicals that prevent consumption by an herbivore. At large scales, secondary metabolites can impact the structure and function of ecosystems, such as when they hinder the decomposition of leaf litter or alter successional patterns (Hunter, 2016). Understanding the ecological functions of secondary metabolites produced by organisms has been one of the main challenges and goals of researchers in the field of chemical ecology. This challenge is increased by these compounds often having more than one function (Anderson et al., 2012; Cembella, 2003), as illustrated by phytoplankton, in which the same molecules have allelopathic properties (effective against competitors), defend against predators, and are used for communication (Hay, 2009; Ianora et al., 2011; Schatz et al., 2007). Moreover, secondary metabolites can be co‐opted by other organisms, such as the sequestration of chemical compounds by invertebrates that makes them unpalatable to predators (Opitz & Müller, 2009; Taboada et al., 2013). Co‐opted secondary metabolites can also provide defences against parasites. This has been well‐studied in terrestrial ecosystems, where there are many examples of secondary metabolites that defend hosts from parasites (as reviewed in de Roode et al., 2013). However, even within animal systems in which animal self‐medication has been well studied, we often do not understand the mechanisms underlying the parasite protection provided by dietary secondary metabolites (Annoscia et al., 2017; Gowler et al., 2015; Lefèvre et al., 2010). Understanding how animals are impacted by secondary metabolites in their food, including how those compounds influence disease risk, is necessary to better predict the outcomes of host‐parasite interactions, the emergence of epidemics, local adaptation, and host‐parasite co‐evolution.

Cyanobacteria produce a wide array of secondary metabolites that have a variety of functions. Among those secondary metabolites, cyanopeptides have been recognized as potent inhibitors of key metabolic enzymes that target serine proteases (von Elert et al., 2005) and protein phosphatases (Schwarzenberger, 2022). There has been extensive research on the impact of cyanobacteria secondary metabolites on aquatic grazers‐particularly on *Daphnia*, which are the dominant grazers in many lakes and ponds, with large impacts on ecosystem functioning, including water clarity, nutrient cycling, and energy flow (Lampert & Sommer, 2007). In nature, *Daphnia* are exposed to a diverse range of phytoplankton that vary both in nutrient quality and secondary metabolites, including *Microcystis aeruginosa*. There has been substantial focus on the consequences of consuming *Microcystis* by *Daphnia*, including studies focusing on toxin‐producing and non‐toxin‐producing strains (e.g., Asselman et al., 2014; Barros et al., 2001; DeMott et al., 1991; Haney, 1987; Lampert, 1981, 2006; Oberhaus et al., 2007; Rohrlack et al., 1999; Wilson et al., 2006). These studies suggest that compounds other than microcystin toxins are key drivers of toxicity and that toxicity manifests by reducing ingestion and digestion rates in *Daphnia* (Lampert, 1981; Lürling, 2003; Rohrlack et al., 2001; Schwarzenberger et al., 2012).

While the impact of cyanobacterial secondary metabolites on *Daphnia* fitness has been extensively studied, these studies have generally focused only on *Daphnia* as grazers. In nature, however, *Daphnia* also hosts a wide variety of parasites (Ebert, 2022). Less is known about how toxins or other secondary metabolites produced by cyanobacteria affect parasitized *Daphnia* and what implications this may have for host‐parasite dynamics in nature. One reason to expect that cyanobacterial secondary metabolites might influence *Daphnia*‐parasite interactions is because the ability of cyanobacteria to produce some of these peptides arose prior to the evolution of the Metazoa (Rantala et al., 2004), which suggests that these compounds evolved originally for purposes other than defense against herbivory. One hypothesis is that these toxins evolved as a result of competition with other microbes (Rohrlack et al., 2013; Schatz et al., 2007). For example, it has been hypothesized that oligopeptides in cyanobacteria can restrain microbial enemies, such as chytrid fungi, by inhibiting proteases produced by the fungus that are used in the host cytoplasm to extract nutrients (Rohrlack et al., 2013). Given this, the secondary metabolites produced by cyanobacteria might also influence *Daphnia*‐parasite interactions. Prior work has revealed that cyanobacterial diets influence the transmission, reproduction, and virulence of parasites in *Daphnia*. For example, *Daphnia* that feed on cyanobacteria while being exposed to the common and virulent fungal parasite *Metschnikowia bicuspidata* are protected from infection (Manzi et al., 2019; Penczykowski et al., 2014; Sánchez et al., 2019). However, more recent work demonstrates that this effect is not due to direct effects of cyanobacterial toxins, including microcystin, on fungal transmission stages (Sánchez et al., 2023). Combined, these results point to an aspect of the interaction inside the host gut that drives the protective effect of cyanobacterial diets.

Global climate change and other anthropogenic impacts (especially eutrophication) are already impacting the frequency and occurrence of harmful algal blooms (HABs) caused by cyanobacteria such as *Microcystis aeruginosa* (Huisman et al., 2018; Pörtner et al., 2022). These blooms are predicted to continue to increase in the future, becoming more intense and more widespread (Smucker et al., 2021). This means that the impacts of cyanobacteria such as *Microcystis* on herbivores – both the negative ones that have typically been the focus of study and positive ones such as protection from infection – will also increase. This is likely to have consequences for biodiversity and ecosystem functioning in aquatic ecosystems, but predicting the direction and strength of the effect is complicated. In order to better predict the impact, we need to both understand the mechanisms underlying the impacts of cyanobacteria on herbivores and understand the potential variation in outcomes (e.g., due to intraspecific variation).

Here, we explore a potential mechanism by which *Microcystis* diets might inhibit infections by the parasite *Metschnikowia bicuspidata* in *Daphnia*. Specifically, we hypothesize that diets that contain protease inhibitors capable of inhibiting gut enzymes such as trypsins and chymotrypsins hinder *Metschnikowia*'s ability to infect a host. *Daphnia* become infected by this fungal parasite after consuming transmission spores (Metschnikoff, 1884; Stewart Merrill & Cáceres, 2018). These transmission spores are contained within a structure called an ascus that then needs to open to release the ascospore (transmission spore; Lachance et al., 1976). The ascospore in *Metschnikowia* species is needle shaped, being pointed on either one or both ends (Lachance, 2011). The specific fungal species that infects *Daphnia, M. bicuspidata*, has spores that are pointed on both sides (hence the ‘bicuspidata'). These needle‐like spores are then able to pierce through the gut of the animal and begin the infection process that results in the reproduction of the parasite in the hemolymph (Stewart Merrill & Cáceres, 2018). The mechanism by which these ascospores are released from the ascus has largely been overlooked in this system, but Metchnikoff suggested that digestive enzymes in *Daphnia* might be responsible for the removal of asci (Metschnikoff, 1884). Some evidence exists for other parasitic species of *Metschnikowia*, that gut enzymes from their host might aid in the removal of the ascus (Lachance et al., 1976; Talens et al., 1973).

**FIGURE 1:**
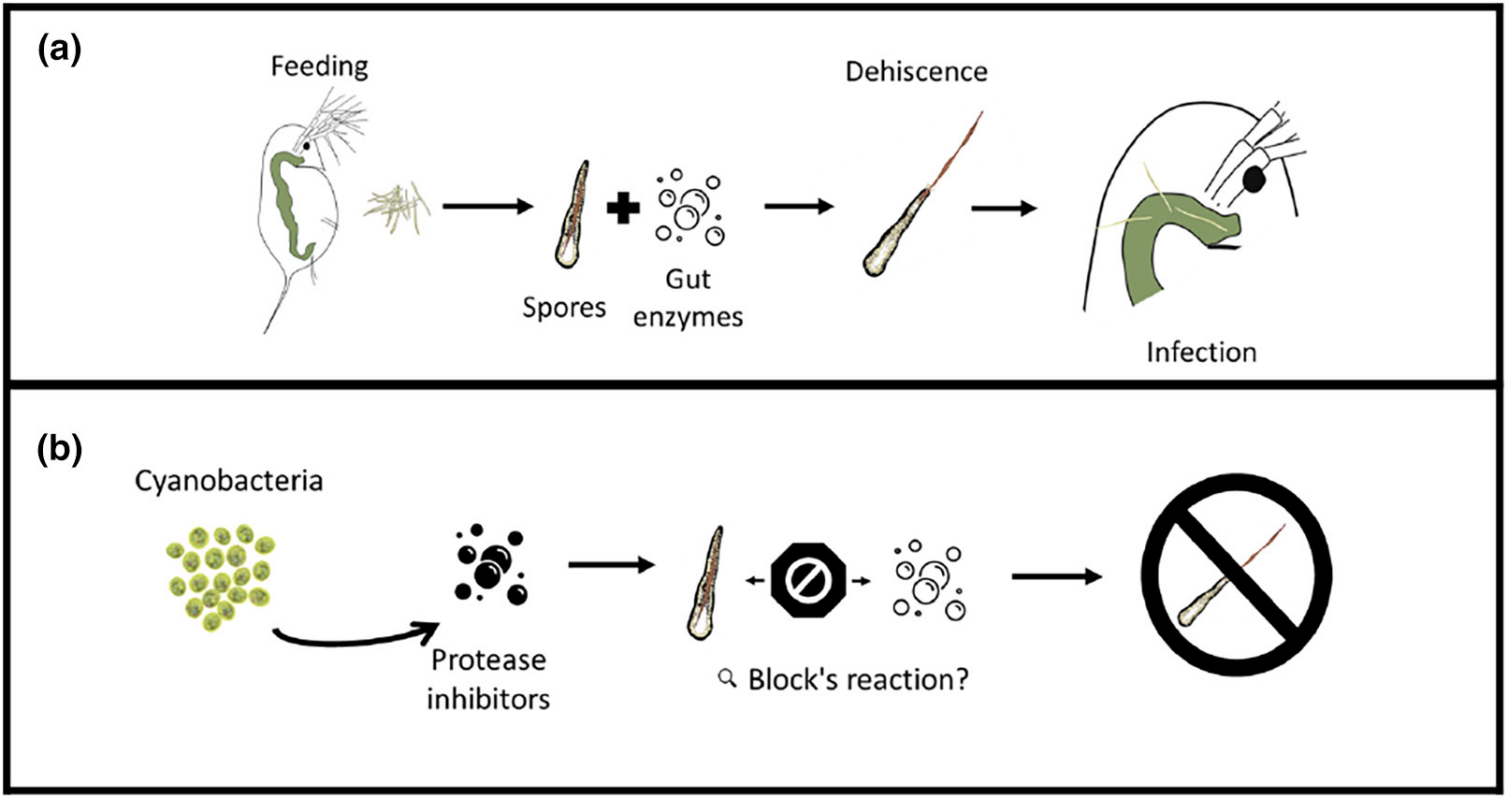
Proposed mechanism of infection for the parasite *Metschnikowia bicuspidata* and proposed mechanisms of how cyanobacteria inhibit infection in this system. In (a), the host feeds on fungal spores in the absence of cyanobacteria. Gut enzymes then trigger the dehiscence of the Metschnikowia spore, which is able to puncture the gut wall, initiating infection. In (b), cyanobacteria produce protease inhibitors that block chymotrypsin, suppressing dehiscence. In this case, the spore is not able to puncture the gut wall, preventing infection.

Because some secondary metabolites produced by *Microcystis* and other cyanobacteria affect *Daphnia* digestive enzymes and because digestive enzymes influence spore morphology, we hypothesized that the effects of cyanobacteria diets on digestive enzymes might influence the infection process. In particular, protease inhibitors produced by cyanobacteria have been shown to inhibit trypsin and chymotrypsin enzymes in the gut of *Daphnia magna* (Agrawal et al., 2005; Schwarzenberger et al., 2010, 2021). We hypothesized that protease inhibitors produced by *Microcystis aeruginosa* inhibit the enzymes responsible for the breakdown of *Metschnikowia* ascus, inhibiting the parasite's ability to infect. Specifically, we hypothesized that when *Daphnia* consume *Microcystis*, protease inhibitors produced by *Microcystis* inhibit digestive proteases in the *Daphnia* gut. Due to inhibition of these enzymes, the ascus is not removed or degraded and therefore the ascospore, unable to be released, is not able to pierce the gut in order to start an infection (Figure 1). In order to address this hypothesis, we ran two experiments that addressed the following questions: (1) Are gut enzymes necessary for dehiscence in the *Daphnia‐Metschnikowia* system (experiment 1), and (2) Do *Microcystis* diets with different protease inhibitors have different impacts on infection (experiment 2)?

## 2 METHODS

### 2.1 Host‐parasite system

Our study used 11 clones of *Daphnia magna*, which is a common species in ponds and lakes, especially in Europe. These clones come from different populations and have been used in studies focusing on the impact of cyanobacterial diets on *Daphnia* gut digestive enzymes (e.g., Schwarzenberger et al., 2021; see Supplementary Material and Table S1 for additional information about these clones). We used the common fungal parasite *Metschnikowia bicuspidata* (‘Standard’ isolate, originally isolated from Baker Lake in Barry County, Michigan). *Daphnia* become infected after consuming transmission spores they encounter in the water column when feeding. Infection takes place when the needle‐shaped spore crosses the gut barrier and is not fought off by a host haemocyte response (Metschnikoff, 1884; Stewart Merrill & Cáceres, 2018). Once infection has taken hold, the fungus replicates within the hemolymph of the host (Stewart Merrill & Cáceres, 2018). The parasite reduces the fecundity and lifespan of infected hosts (Auld et al., 2012). *Metschnikowia* is an obligate killer, meaning it must kill its host in order to transmit to a new host (Ebert, 2005); transmission spores are released into the environment after host death, after which they can be consumed by a new host, completing the parasite's life cycle.

### 2.2 Cultivation and preparation of phytoplankton food

We cultivated five strains of phytoplankton: one green alga and four cyanobacteria. We obtained the green alga *Scenedesmus obliquus* (UTEX 3155) from the Culture Collection of Algae at the University of Texas at Austin. The cyanobacteria *Microcystis aeruginosa* strain CYA160/1 and strain CYA43 came from the Norwegian Institute for Water Research. Finally, the *Microcystis aeruginosa* strains wild type (WT) PCC7806 and mutant (MT) PCC7806 ‐mycB were obtained from the Pasteur Culture Collection of Cyanobacteria at the Institute Pasteur. The *Microcystis* strains used here vary in their production of microcystin and also their protease inhibitors (some effective at inhibiting chymotrypsin and others at inhibiting trypsin in the gut of *Daphnia* (Agrawal et al., 2005; Schwarzenberger et al., 2021)). We used the CYA43 strain of *Microcystis* because it strongly inhibits chymotrypsin activity in *D. magna* (von Elert et al., 2012), but does not produce microcystins. We used the CYA160 strain because a prior study found it protected *Daphnia dentifera* against infection by *Metschnikowia*, yielding much lower levels of infection than *Scenedesmus* (Sánchez et al., 2019). We used the PCC7806 and PCC7806‐mycB strains to allow us to explore variation among *Microcystis* strains in their effects on the infection process and to isolate whether there is an effect of microcystin, since PCC7806‐mycB is a microcystin‐deficient mutant of the wild‐type strain (Dittmann et al., 1997); hereafter, we refer to these strains as PCC7806‐WT (for the wild type) and PCC7806‐MT (for the microcystin‐deficient mutant type).

We grew all cultures in chemostats with 24‐h light. *Scenedesmus* was grown in standard COMBO media, *Microcystis* CYA43 and CYA160 were grown in Z8 media, *Microcystis* PCC7806‐WT in BG11 media, and *Microcystis* PCC7806‐MT was grown in BG11 media with 5 μg/mL chloramphenicol (which is necessary to keep microcystin genes inactivated; Dittmann et al., 1997). Food for the experiments was prepared weekly. We harvested cells in 50 ‐mL Falcon tubes and centrifuged (Sorvall St 16, ThermoScientific) for 10 min at 1690 *g*. After, we decanted media from the tubes and resuspended cells in ADaM (Klüttgen et al., 1994). The volume was adjusted to a concentration of 100 mg C/L and this solution was used to feed animals at a final concentration of 2 mg C/L. *Microcystis* PCC7806‐MT was rinsed and spun down twice with milliQ water before resuspending in ADaM to rinse out any chloramphenicol that may have been left from growth media.

### 2.3 Experiment 1. Dehiscence assay: Isolating the impact of gut extracts and chymotrypsin on spore dehiscence

To test whether diet impacted dehiscence of *Metschnikowia* spores, we exposed them to extracts from the guts of animals that were fed *Scenedesmus* or from the guts of animals that were fed *Microcystis* diets that are known to inhibit chymotrypsin activity in the gut. If chymotrypsin promotes spore dehiscence, we expected higher levels of dehiscence for spores exposed to gut extracts from *Scenedesmus* (which should have normal chymotrypsin activity) than from *Microcystis*. To further test the impact of chymotrypsin, we also added pure chymotrypsin (CAS 9004‐07‐3, Sigma‐Aldrich) to the gut extracts of animals fed *Microcystis*, to see if we could recover the levels of dehiscence seen for spores exposed to gut extracts from animals fed green algae. This assay was done using extracts from the guts of the ‘May 20’ *Daphnia magna* clone because prior work indicated the susceptibility of this clone differed greatly between *Scenedesmus* and *Microcystis* diets.

We reared 5 individuals of clone ‘May 20’ per 150‐mL beaker filled with 100 mL of filtered lake water, feeding them 2 mg C/L *Ankistrodesmus falcatus* AJT strain (Schomaker & Dudycha, 2021; Tessier et al., 1983) until they were 5–6 days old. At this time, we moved the animals to clean filtered lake water, keeping 5 individuals per beaker, and fed them 2 mg C/L of either *Scenedesmus* or *Microcystis aeruginosa* CYA43; we had 20 replicate beakers for the *Scenedesmus* treatment and 40 replicate beakers for the *Microcystis* treatment. We had twice the number of *Microcystis* treatment replicates because we needed extra animals to make gut extracts, 100 of these animals would be used for the regular gut extract treatment, and 100 would be used for the gut extract + chymotrypsin treatment, as described below. On the next day, when animals were 6–7 days old, we again fed them 2 mg C/L of their treatment diet. On the following day, we placed animals in 2‐mL Eppendorf tubes, placing 20 animals per tube (combining four of the beakers into a single tube) for a total of 5 tubes with 100 animals total for the *Scenedesmus* diet treatment and 10 tubes with 200 animals total for the *Microcystis* treatment, and stored the tubes with animals at −20°C. We later dissected the entire guts of animals and placed the 20 guts in a tube, again for a total of 5 tubes with 20 guts each for the *Scenedesmus* diet treatment and 10 tubes with 20 guts each for the *Microcystis* diet treatment. We added 100 μL of 0.1 M potassium‐phosphate (P‐P) buffer pH 7.5 to each of the tubes and homogenized them using a pestle. We centrifuged the tubes at 14,000 *g* for 3 min, then transferred the supernatant into a 2 mL Eppendorf tube. We used bovine chymotrypsin (Sigma Aldrich) as a positive control, resuspending it in P‐P buffer to a concentration of 10% w/v. Finally, we used P‐P buffer as our negative control.

We tested the impact of these gut extracts (or controls) on parasite transmission spores. Specifically, we measured how many spores lost their ascus over time. The extracts and the concentrations we tested are the following: 5% w/v *Scenedesmus* gut extract, 5% w/v *Microcystis* gut extract, 5% w/v *Microcystis* gut extract with 1% w/v bovine chymotrypsin, 1% w/v bovine chymotrypsin, and finally, P‐P buffer as a negative control. We predicted that, if chymotrypsin is important for dehiscence, the highest level of dehiscence (that is, most spores without asci) would occur in treatments with high levels of chymotrypsin activity, and the lowest levels would be in treatments with low activity levels of chymotrypsin (inhibited due to diet). More specifically, we predicted that spores incubated in gut extracts of animals fed *Microcystis* would have lower levels of dehiscence than those incubated in gut extracts of animals fed *Scenedesmus*, and that there would be high levels of dehiscence of spores incubated in pure chymotrypsin or in those where chymotrypsin was added to gut extracts of animals fed *Microcystis*.

To test this, we placed 250 μL of spore slurry containing ~100,000 spores of the fungal parasite *Metschnikowia bicuspidata* in wells of a 96 well plate. We did this for 20 wells placed randomly throughout the plate. One person would place a treatment picked haphazardly to one of the wells in the plate and note the time at which the treatment was placed. After 30 and 210 min, another person would count the number of spores with and without asci within a given well without knowing which treatment was placed in that well. The counts were done under a compound microscope at 400× with a Neubauer counting chamber. This was repeated for every treatment for a total of 4 replicates per treatment.

### 2.4 Experiment 2. Quantification of effects of diet treatments on gut digestive enzymes and infection levels

Because susceptibility and tolerance to protease inhibitors are *Daphnia* clone‐ and diet‐dependent, we wanted to measure variation in protease activity on different diets and test how these gut enzyme levels corresponded with host susceptibility and parasite fitness. To quantify the impact of diet on gut proteases, we measured the trypsin and chymotrypsin activity of gut extracts from animals fed different diets. We also measured variation in infection outcomes for animals fed on these different diets. We were interested in both the variation in gut protease activity and in whether reduced protease activity correlated with decreased infection.

We ran a factorial experiment with 11 *Daphnia* clones and 5 diets. We ran this experiment in 7 blocks, aiming for 5 replicates per clone × diet treatment per block. We interspersed blocks that were used to measure enzyme activity and those used for infection assays. Blocks 1–3 were used entirely for infection assays, blocks 4 and 6 entirely for enzyme assays, and blocks 5 and 7 were used for both infection assays and enzyme activity; for blocks 5 and 7, we aimed for 10 replicates per treatment, and half of those replicates were used for the enzyme activity assay. While we aimed for 5 replicates per block, in some cases, we lost animals, especially due to cyanobacteria toxicity; in these cases, we tried to replace the replicate in a future block. This was particularly problematic in blocks 1 and 2. For the two PCC7806 diet treatments, very high mortality means that the dataset does not include any animals from blocks 1 and 2 for these treatments in our analyses.

We first reared individuals of each clone under standardized lab conditions for multiple generations prior to the experiment. For all blocks, neonates (0–1 day old) were harvested from mothers and placed 5 each in 150 ‐mL beakers with 100 mL of filtered lake water. Neonates were fed *Ankistrodesmus falcatus* daily for 5 days. When juveniles were 5–6 days old, each juvenile was placed individually in a 50 ‐mL beaker with 30 mL of filtered lake water. Juveniles were fed their corresponding treatment diet as follows: 2 mg/L C of *Scenedesmus*, 2 mg/L C of *Microcystis* CYA160, 2 mg/L C of *Microcystis* CYA43, 1 mg/L C *Microcystis* PCC7806‐WT with 1 mg/L C *Scenedesmus*, and 1 mg/L C *Microcystis* PCC7806‐MT with 1 mg/L C *Scenedesmus*. For the two PCC7806 treatments, we needed to use a 50:50 mix of the treatment diet:*Scenedesmus* to promote survival. The first two blocks of the experiment used 100% of the *Microcystis* strains, but the very high mortality meant we could not use those animals in the experiment. Therefore, for blocks 3–7, animals in these two treatments were fed a 50:50 mix of the *Microcystis* strain and *Scenedesmus*.

For animals in blocks 4 and 6 and for the half of blocks 5 and 7 animals that were used to measure protease activity, on the next day (that is, when animals were 6–7 days old), we transferred animals to clean beakers filled with 30 mL of fresh filtered lake water and fed them half the amount from the previous day of their corresponding treatment food; this was done to be consistent with the treatment of animals for the infection assays (see below). After 24 h, we sacrificed these animals, preserved them in Eppendorf tubes with no water and stored them at −20°C. At a later date, animals were grouped to have 20 animals per clone × diet treatment (for a given clone × diet treatment combination, animals from blocks 4 to 7 were combined into one tube) in a given tube and used to measure the proteolytic activity of trypsin and chymotrypsin. Trypsin activity was assayed using the substrate N‐benzoyl‐ DL‐arginine p‐nitroanilide (BapNA), while chymotrypsin activity was assayed using N‐succinyl‐L‐alanyl‐L‐alanyl‐L‐propyl‐L‐phenylalanine 4‐nitroanilide (SucpNA). Detailed methods used to quantify potential enzyme activity (as ΔmAU/min/μg protein content) are given in the supplement.

For the five infection assay blocks, when animals were 6–7 days old, we transferred them individually to clean beakers filled with 30 mL of fresh, filtered lake water. On this day, all animals were exposed to 500 spores/mL of *Metschnikowia* and fed half the amount from the previous day of their corresponding treatment food; prior work has shown that reducing phytoplankton exposure promotes spore uptake by hosts. After 24 h, we placed each animal in a 150‐mL beaker with 100 mL of filtered lake water and fed each animal their corresponding treatment diet with the original amount of 2 mg C/L but 50:50 their assigned treatment diet:*Scenedesmus*. We checked for mortality daily and counted offspring twice a week during water changes, removing offspring from the experiment. Animals were fed their treatment diets mixtures daily. Animals that died throughout the experiment were preserved in 100 μL of MilliQ water and stored at 4°C for later spore counts. At the end of 20 days, any remaining animals were preserved in 100 μL of MilliQ water and stored at 4°C for later spore counts. We determined the final abundance of transmission spores in hosts by counting spores under a compound microscope at 400× using a Neubauer counting chamber. Animals were diagnosed as infected if they contained transmission stages (equivalent to ‘terminal infection’ in Stewart Merrill et al., 2019 and to ‘effective infection’ in Dziuba et al. (2024)).

### 2.5 Analyses

All analyses were done in R (v 4.0.3). For all analyses, we checked data for normality using the Shapiro test; if data were not normal, we adjusted the analysis as described for individual analyses below. For generalized linear mixed models with non‐normal distributions we also checked for overdispersion.

We were interested in whether the degree of spore dehiscence varied based on the gut extract or control to which they were exposed, with a prediction that there would be more spores without asci in treatments with chymotrypsin. To test this, we analysed the number of spores without asci per well, with four replicate wells per gut extract or control treatment (Table 1). For the gut extract treatments, the extracts from one tube containing the guts of 20 animals (see above) were added to a single well. Because the data were not normally distributed and because there were no random factors or interactions in the model, we used a Kruskal‐Wallis test to evaluate differences in the number of spores without dehiscence across the different gut extracts and controls. We then used the compare_means function from the ggpubr package (Kassambara & Kassambara, 2020) to compare specific treatments. We were particularly interested in the comparison of dehiscence in gut extracts from animals fed *Scenedesmus* versus *Microcystis* and in treatments with added chymotrypsin.

**TABLE 1:** Replication information.

Scale of inference	Scale at which the factor of interest is applied	Number of replicates at the appropriate scale
**Effects of gut extracts on spore dehiscence (Figure** **2** **)**		
Comparison of dehiscence of parasite spores after exposure to different extracts or compounds	Wells in a 96 well plate	4 replicates per treatment: guts from host fed *Scenedesmus*, guts from host fed *Microcystis* CYA43, guts from host fed *Microcystis* CYA43 plus pure chymotrypsin, chymotrypsin only, and negative control of P‐P buffer only; for the chymotrypsin and negative control treatments, the compound was applied to each well individually; for the treatments with gut extracts, one tube of 20 animals was used for a single well, with different tubes used for the replicate wells within a treatment.
**Enzyme activity assay (Figure** **3** **)**		
Comparison of enzyme activity of gut extracts from hosts fed different diets	Individual animals from each clone were exposed to a given diet individually; animals were then pooled by genotype × diet combination prior to the enzyme activity assay in order to have enough individuals to do the assay.	There was one tube per clone × diet combination, with technical replicates within those combinations. The same tube of pooled animals was used for the trypsin and chymotrypsin enzyme activity assays. Note: the analyses presented in the paper include technical replicates for each diet x clone combination; if we run the analysis with the average of those technical replicates, the results are qualitatively the same.
**Proportion infected on different diets (Figure** **4** **)**		
Comparison of clones and diets	Individual host	4–14 per diet × clone combination (mean: 10.3, median: 10)
**Change in infection for *Microcystis* CYA43 versus *Scenedesmus* (Figure** **5** **)**		
Comparison of clones fed two diets	Individual animals within a clone × diet treatment combination; animals within a clone were then pooled for this analysis to get an overall proportion infected on a given diet	11 clones

To evaluate differences in protease activity among diets across all clones, we measured the level of enzyme activity for pooled animals from a given clone by diet treatment combination (Table 1). We then used a generalized linear mixed model (GLMM) using a gamma distribution with the change in absorbance units per minute per total amount of protein as the response variable, diet as a fixed effect, and clone as a random effect. The gamma distribution was identified using the fitdist function of the fitdistrplus package in R (Delignette‐Muller & Dutang, 2015). In this analysis, we had technical subreplicates within a diet × clone combination; a model that included just the average value of these technical subreplicates (i.e., one value per diet x clone combination) yielded the same qualitative patterns. Because trypsin and chymotrypsin levels are two different phenotypes that can respond independently, we did this analysis separately for the two enzymes, trypsin and chymotrypsin, but the same tubes of animals were used for the assays for the two enzymes.

We were interested in whether diet influenced the susceptibility of hosts of different clones, predicting that infection prevalence would vary across diets. This analysis included data collected on individual animals that were exposed to spores and a particular diet. Prior to analysis, we removed the following from the dataset: 44 males as well as 19 animals that died prior to day 7 post‐parasite exposure because we could not diagnose these animals as infected or not. After excluding these animals, we had 565 animals in our analysis; there were 4–14 (mean: 10.3, median: 10) individuals per diet x clone combination (Table 1). We then used a GLMM to evaluate differences in infection prevalence among diets. The model used a binomial family distribution with infection status (0 = no, 1 = yes) as the response variable, diet as a fixed effect, and both block and clone as random variables. Next, to evaluate how each diet impacted infection among clones, for each clone, we first calculated an unstandardized effect size of consuming a given cyanobacterial diet versus the control green diet (calculated as the proportion infected on the cyanobacterial diet –proportion infected on the *Scenedesmus* diet); for this analysis, each clone mean was a replicate (Table 1). In this analysis, a cyanobacterial diet that protects against infection relative to the control diet would have a value less than 0. We anticipated that protective diets would have the biggest impact on clones that were highly susceptible to the control diet. Finally, we calculated the regression of the proportion infected of a given clone on the control diet *Scenedesmus* (which indicates the overall susceptibility of the clone) and the proportion infected by a given strain of *Microcystis*; for this analysis, each clone mean was a replicate (Table 1). Because clones vary in their susceptibility, with some being broadly more susceptible than others, we anticipated that these correlations would be positive, and that protective diets would be indicated by regressions with a slope substantially less than 1 (which would indicate lower susceptibility on the *Microcystis* diet than on *Scenedesmus*).

## 3 RESULTS

### 3.1 Chymotrypsin promotes dehiscence

**FIGURE 2:**
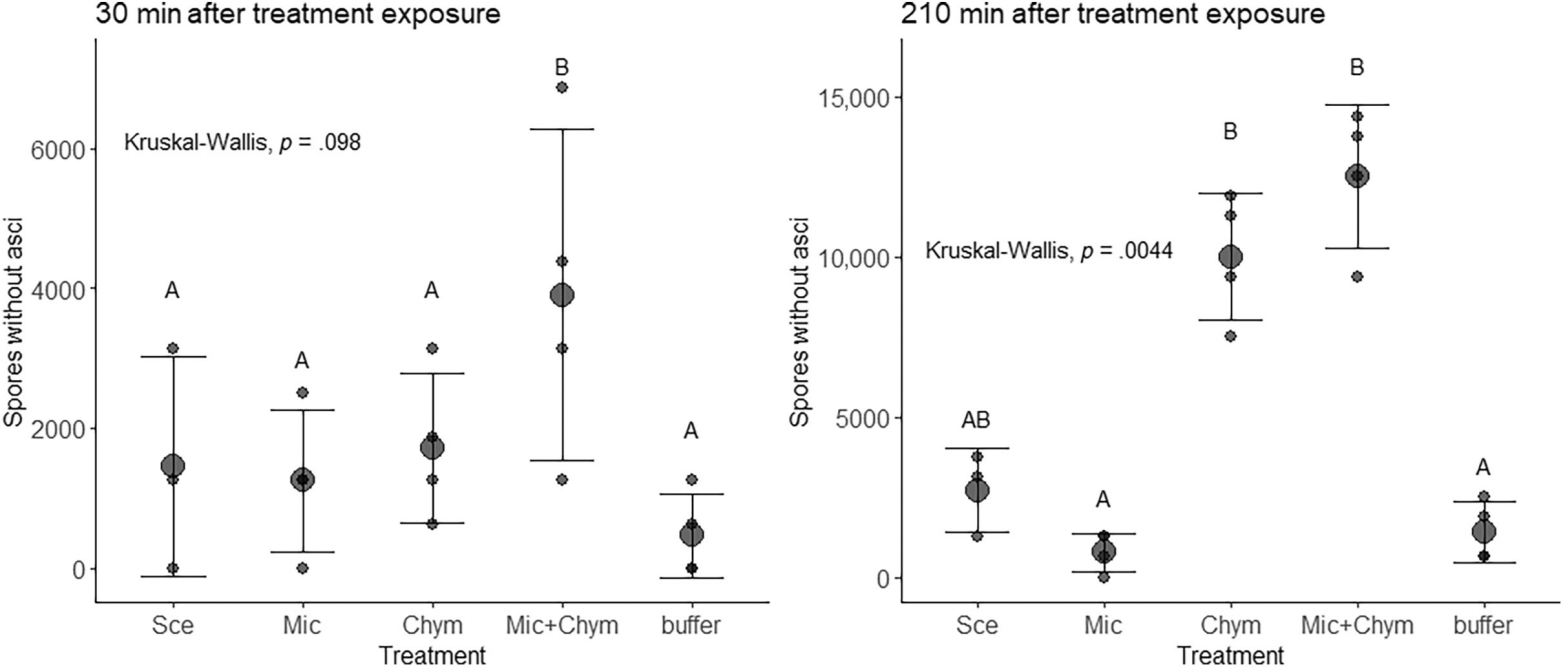
Chymotrypsin promotes the dehiscence of fungal spores. The treatments included extracts of the guts of animals fed *Scenedesmus* (‘Sce’) or *Microcystis* (‘Mic’), pure chymotrypsin (‘Chym’), chymotrypsin added to the gut extracts from animals fed *Microcystis* (‘Mic+Chym’) and a buffer control. Large circles represent mean in each treatment while small circles represent individual data points. Bars represent standard error.

Treatments in which spores were exposed to chymotrypsin had more spores without asci (Figure 2). While there was only a marginal overall effect of treatment on dehiscence after 30 min (*χ* ^*2*^ = 7.8, df = 4, *p* = .098), there was a strong effect of treatment on dehiscence after 210 min (*χ* ^*2*^ = 15.16, df = 4, *p* = .004; Figure 2, Table 2). We were particularly interested in whether dehiscence was higher for extracts from animals fed *Scenedesmus* versus *Microcystis* and whether chymotrypsin increased dehiscence. After 210 min, there was a marginally significant lower number of spores without asci in treatments with gut extracts from animals fed *Microcystis* (*p* = .099) compared to treatments where guts came from animals fed *Scenedesmus*. When chymotrypsin was added to gut extracts from animals fed *Microcystis*, the number of spores without asci increased substantially (*Microcystis* vs. *Microcystis* + chymotrypsin: *p* = .029), indicating that chymotrypsin promotes dehiscence.

**TABLE 2:** Statistical results from the analyses corresponding to Figures 2 through 5.

Effect of gut extracts on spore dehiscence (Figure 2)			
Kruskal‐Wallis	df	χ^2^	*p*‐value
Treatment‐30 min	4	7.8261	.09816
Treatment‐210 min	4	15.163	.0044

Enzyme activity assay (Figure 3)				
	npar	AIC	LRT	*p*‐value (Chi)
GLMM‐trypsin				
Diet	4	606.18	494.37	<2e‐16*
Random effects	**Variance**	**SD**		
Clone	0.02112	0.1453		
Residual	0.40266	0.6346		
GLMM‐chymotrypsin				
Diet	4	1144.09	580.47	<2.2e‐16*
Random effects	**Variance**	**SD**		
Clone	0.00345	0.05874		
Residual	0.25298	0.50297		

Infection global analyses – GLMM (Figure 4)				
Diet	4	612.86	24.602	6.049e‐05*
Random effects	**Variance**	**SD**		
Clone	1.0359	1.0178		
Block	0.3545	0.5954		

Analysis of change in infection for *Microcystis* CYA43 versus *Scenedesmus* (Figure 5)				
Linear model	Estimate	SE	*t*‐value	*p*‐value
Prop Sce infected	0.50967	0.13082	3.896	.00364*
	***r*** ^**2**^	0.5864	***F*‐statistic**	15.18

*Note*: Asterisks (*) indicate significant p‐values.

### 3.2 Microcystis aeruginosa CYA43 decreases chymotrypsin activity in daphnia

**FIGURE 3:**
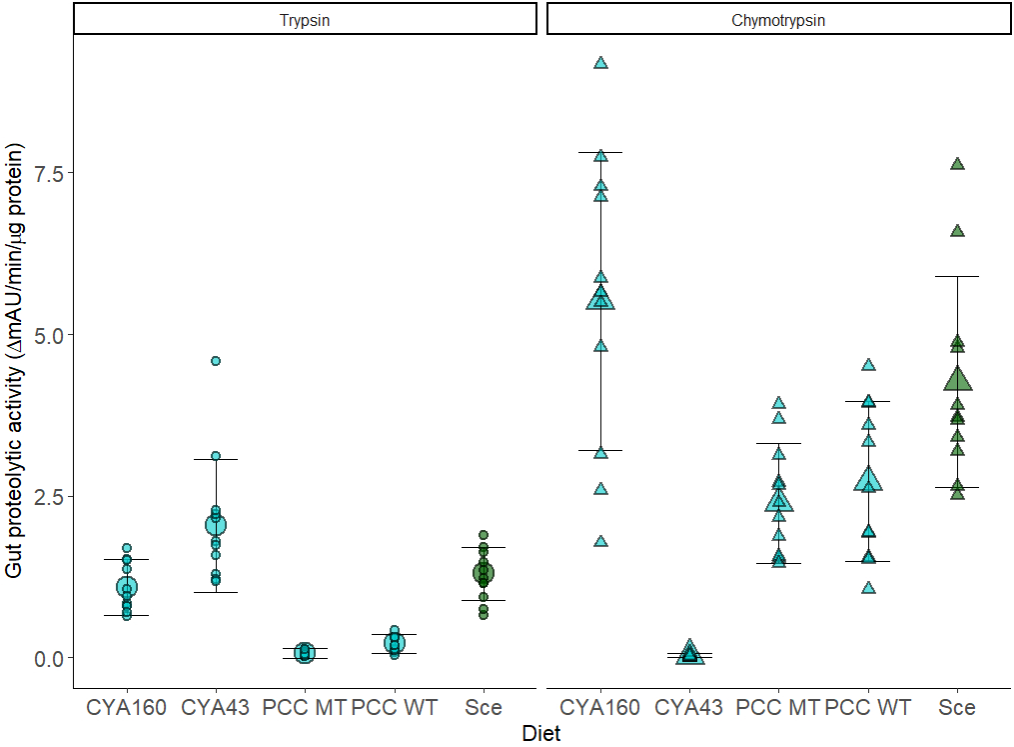
Gut protease activity of *D. magna* clones 7–8 days old on different treatment diets. Large circles and large trianlgles are means across clones, while small circles and small triangles are means of a given clone. Error bars represent standard deviation of mean.

Diet influenced trypsin and chymotrypsin activity in *Daphnia* (Figure 3, Table 2), but in different ways across the different *Microcystis* strains. Animals fed *Microcystis* strains PCC7806‐WT and PCC7806‐MT had lower levels of trypsin activity compared to animals fed *Scenedesmus* (PCC7806‐WT vs. *Scenedesmus*: *t* = 11.5, *p* < .0001, PCC7806‐MT vs. *Scenedesmus*: *t* = 12.6, *p* < .0001), and those fed *Microcystis* CYA160 had marginally significantly lower trypsin levels (Figure 3 left panel; CYA160 vs. *Scenedesmus*: *t* = 1.77, *p* = .076). In contrast, animals fed CYA43 had significantly higher trypsin levels compared to those fed *Scenedesmus* (*t* = −4.28, *p* < .0001). Chymotrypsin activity in all clones was different from the control diet *Scenedesmus* (Figure 3 right panel). Most notably, *Microcystis* CYA43 decreased chymotrypsin activity significantly compared to other diet treatments (*t* = 14.2, *p* < .0001).

### 3.3 Lower infection levels in animals fed *Microcystis aeruginosa CYA43*

**FIGURE 4:**
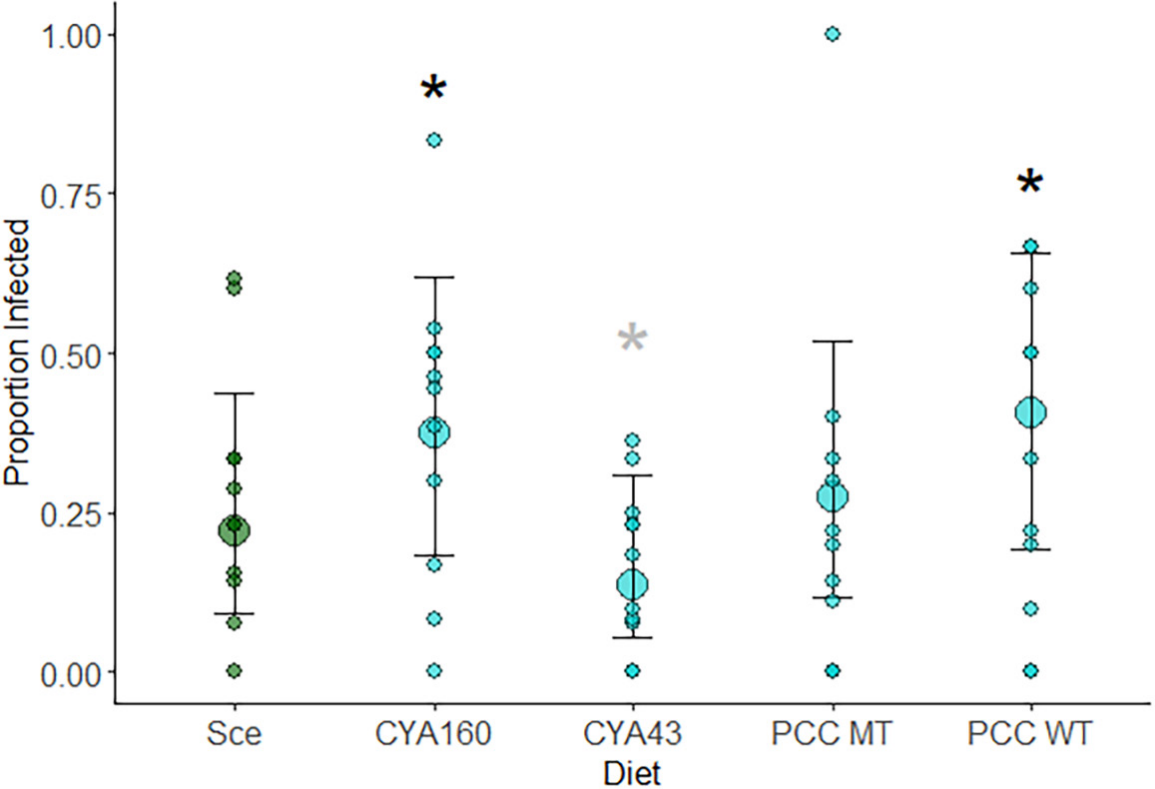
Proportion of infected *Daphnia* individuals in each of the treatment diets. Large circles are means across clones, while small circles represent means for a given host clone. Black asterisks indicate treatments where the proportion infected significantly differed compared to Scenedesmus; the grey asterisk indicates a marginally significant difference (CYA43 vs. Scenedesmus: *Z* = −1.72, *p* = .0849).

There were significant differences in infection levels across the diet treatments (Table 2). Notably, there were qualitative differences among *Microcystis* strains in their effects on infection. Compared to *Scenedesmus* infection levels, two *Microcystis* diet treatments (CYA160 and PCC7806‐WT, both of which produce microcystin) had significantly higher levels of infection, but CYA43 somewhat reduced infection levels (Figure 4).

**FIGURE 5:**
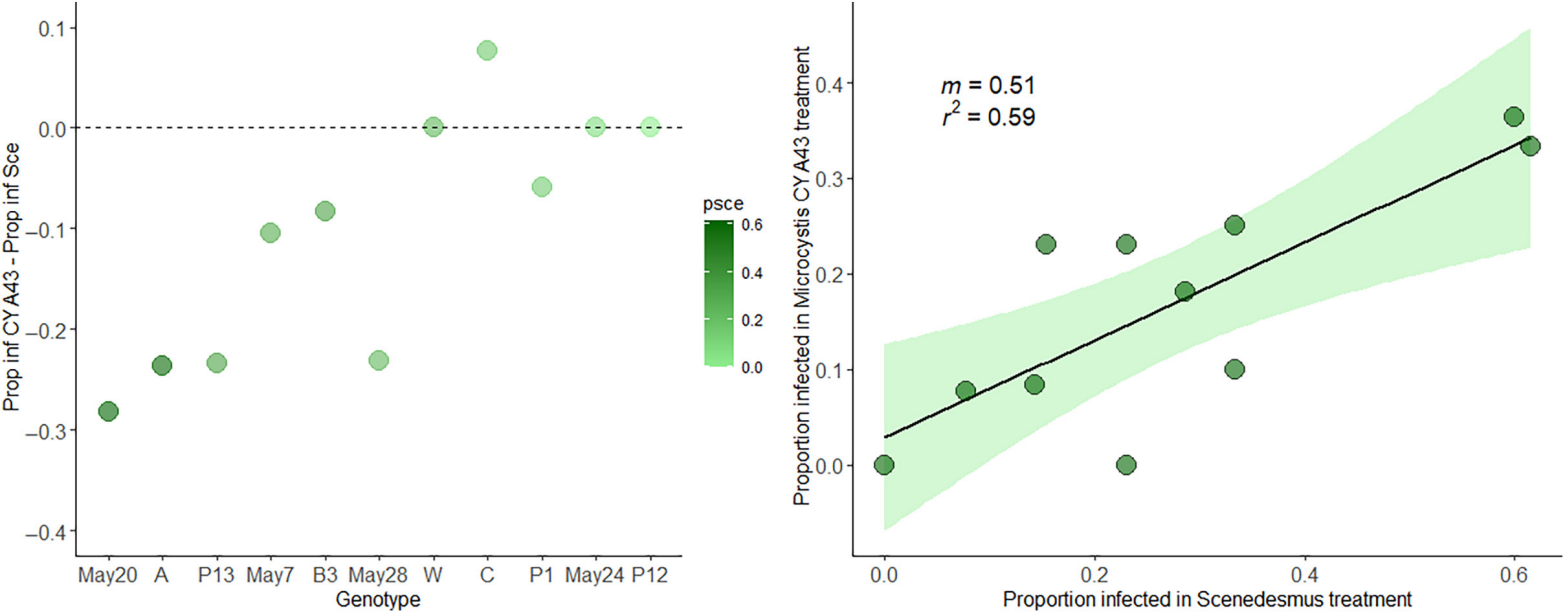
Left panel: Effect size on infection prevalence on *Microcystis* CYA43 diet; clones are arranged from most susceptible to *Metschnikowia* (based on the *Scenedesmus* treatment) on the left side of the x‐axis to those that are least susceptible on the right, with shading corresponding to that susceptibility. Points below the line indicate a lower infection when fed on CYA43. Right panel: Linear regression of proportion infected in the *Scenedesmus* diet vs. proportion infected when fed *Microcystis* CYA43. The slope (*m* = 0.51) indicates that clones that were more susceptible were particularly protected from infection; a slope of 1 would indicate that clones had the same susceptibility when fed CYA43 as when fed *Scenedesmus*.

The above results show that chymotrypsin promotes dehiscence (a necessary step in the infection process), that CYA43 strongly reduces chymotrypsin, and that infection levels were lowest in animals fed CYA43. However, the last effect was only marginally significant, potentially because of relatively low levels of infection in the experiment overall. Looking across host clones (which varied in their susceptibility to infection), most clones had lower infections when fed CYA43 during exposure, as compared to *Scenedesmus* (Figure 5 left panel). This was not the case for the other diets, where many clones had increased susceptibility (as compared to the *Scenedesmus* diet treatment) when feeding on *Microcystis* (Figure S1). Thus, CYA43 tended to reduce infection levels, with the strongest protection for the most susceptible clones (e.g., ‘May20’, ‘A', and ‘P13’). On average, susceptibility on CYA43 was about half that on *Scenedesmus* (slope estimate: 0.51, SE = 0.13; Figure 5 right panel). In contrast, the regressions for the impact of all the diets other than CYA43 tended to have a slope greater than 0.5, in some cases approaching a slope of 1, meaning that there was no decrease in infection prevalence when feeding on those diets (Figure S1; Table S3). This suggests that more susceptible clones may generally benefit from consuming cyanobacteria instead of green algae when challenged by *Metschnikowia* spores, but that the effect varies depending on the strain of cyanobacteria.

In other words, CYA43 had the strongest protective effect against infection in the clones that were most susceptible when fed *Scenedesmus*. In contrast, when fed other diets, there was more variation among clones in whether infection increased or decreased (compared to *Scenedesmus*), and the correlation between susceptibility to *Scenedesmus* vs. when fed *Microcystis* was not significant in most cases (see Supplemental results).

## 4 DISCUSSION

Our study shows that chymotrypsin aids in the dehiscence of the fungal parasite *Metschnikowia*; spores incubated in the presence of chymotrypsin had much higher levels of dehiscence. Moreover, as had been found in earlier studies (Schwarzenberger et al., 2010; von Elert et al., 2012), we found that certain cyanobacterial diets inhibit gut digestive proteases. Notably, given the dehiscence results, *Microcystis aeruginosa* strain CYA43 strongly inhibited chymotrypsin activity. Finally, hosts who consumed CYA43 had reduced infection levels, with the strongest protective effects seen in the most susceptible host clones. Taken together, these results suggest chymotrypsin inhibition by cyanobacterial diets leads to lower dehiscence, which decreases infection success, protecting hosts from this virulent parasite.

We hypothesize that the observed reduction in infection levels for hosts that fed on *Microcystis* CYA43 is probably due to fewer spores puncturing the gut of the host since they have not dehisced. In a separate study using the same strain of cyanobacteria as the diet but for a different species of *Daphnia*, results showed that there were a dramatically lower number of spores puncturing the gut of *Daphnia* that had consumed *Microcystis* CYA43 versus the control diet *Scenedesmus* (M.L. Fearon et al. in prep). This in turn translated into lower infection levels in hosts fed that treatment diet. Unfortunately, we were unable to collect data on the number of spores attacking the gut in this experiment; the larger body size of *Daphnia magna* hosts prevented us from being able to visualize the gut clearly enough to see attacking spores.

*Microcystis* CYA43 strongly reduced chymotrypsin proteolytic activity in all host clones (Figure 3), but there was more variation among host clones in the effects of *Microcystis* CYA43 on host susceptibility. We consider three reasons for this variation: (1) variability in feeding rate (and, therefore, rates of parasite encounter), (2) differences in gut enzyme isoforms, and (3) variation in physical barriers such as gut thickness. Feeding rates can vary in *Daphnia* for various reasons, including body size. It is possible that the clones differed from one another in body size at the time of exposure, but we do not have data on body size, so we cannot evaluate whether this contributed to the variation we observed. Gut protease isoforms might also explain why some clones were not as susceptible to parasites compared to other clones in the same diet. More specifically, some clones might already produce chymotrypsin forms that are less effective at promoting dehiscence, therefore rendering them resistant to parasites regardless of diets. If this is the case, we would not expect a notable change in infection susceptibility when feeding on a diet that inhibits chymotrypsin (a result consistent with the pattern in Figure 5). Finally, gut thickness can act as a physical barrier to infection (Stewart Merrill et al., 2019); in a recent study on *Daphnia dentifera*, animals with the thinnest and thickest gut walls were more resistant to infection (Sun et al., 2023). Thus, even in the absence of cyanobacterial diets, some clones may already be relatively resistant to infection, though we don't have information on gut thickness for these clones. If there is variation in gut thickness across clones, this might mask the effects of diets (which is again consistent with the pattern in Figure 5). Overall, we suggest that chymotrypsin‐triggered dehiscence is an important step towards infection but that other factors also influence the infection process; these can act in combination and likely help explain variation in susceptibility.

Diet influences parasites via more than just gut protease inhibition. We found low within‐host parasite reproduction in one of the cyanobacterial diets (Supplementary Materials, Figure S2), *Microcystis aeruginosa* PCC7806 WT. This cyanobacterium produces microcystin and protease inhibitors that inhibit trypsin in the guts of *Daphnia*. Hosts had low fitness on this diet (Supplementary Materials, Figure S3), which likely drove the low parasite spore yield. Our data suggests this low host and parasite fitness may be due to microcystin toxicity on the host rather than decreased amino acid acquisition from trypsin inhibition. Trypsin inhibition was comparable between the PCC wild type and the mutant strain, with both strains having the lowest *Daphnia* trypsin activity among diets. However, offspring reproduction was lowest in the wildtype PCC strain compared to the mutant strain. This same trend follows the spore production pattern, in which we found low host spore numbers for the wild‐type strain compared to the mutant strain. Thus, it appears that microcystins at least partially drove the negative effects on host and parasite fitness for this diet.

From the host's perspective, the impact of diet on fitness is dependent on the host clone (Schwarzenberger et al., 2021). It also varied substantially across the four strains of *Microcystis aeruginosa* that were studied. While hosts incurred a fitness cost on certain cyanobacterial diets (i.e., Microcystis PCC 7806), when feeding on *Microcystis* CYA43, hosts gained protection against *Metschnikowia* infection without suffering reduced fecundity, even with the decreased chymotrypsin activity in the gut. One thing these results suggest is that trypsin might be more important in the acquisition of nutrients: hosts fed the two PCC diets had low trypsin and low reproduction, whereas hosts fed CYA43 and *Scenedesmus* had high trypsin and high reproduction.

The lack of a fitness cost when fed CYA43 in the absence of the parasite (this study; Lange et al., 2023) and its protective effect against the parasite is also interesting because the criteria for a diet to be medicinal include that the secondary metabolite or compound has to be toxic in the absence of a parasite (de Roode et al., 2013; Singer et al., 2009). Although our study does not address whether *Daphnia* exhibit self‐medication behaviour, *Daphnia* do encounter and feed on cyanobacteria in the water. Our results indicate that some cyanobacteria protect against infection without cost to the host in the absence of parasites. It is possible that there was no cost to cyanobacterial diets because hosts were not fed pure cyanobacteria for the whole study. In the earlier study by Lange et al. (2023), CYA43 was always mixed with a green alga. In the present study, *Daphnia* were only exposed to 100% cyanobacteria for 2 days (before and during parasite exposure); throughout the rest of the experiment, animals were fed a mixture of cyanobacteria and green algae. Our results suggest that the presence of *Scenedesmus* in the food mixture was sufficient in this treatment to keep host fitness comparable to the control diet of 100% *Scenedesmus*. Supporting this, in a different experiment, *Daphnia dentifera* that were fed 100% *Microcystis* CYA43 throughout the study had significantly lower fecundity than those fed 100% *Scenedesmus* (M.L. Fearon et al. in prep). In that study, hosts fed a 50:50 mix of the two phytoplankton had fecundity that was similar to hosts fed 100% *Scenedesmus*. There has been an effort in the field to reconsider what scientists call a nutrient versus a toxin or a medicine (Raubenheimer & Simpson, 2009), suggesting that we based those definitions on the dosage of the compound rather than the particular effect it may have in a given organism. We suggest that we should view diets as falling along a gradient that can range in nutrition, toxicity, and medicinal value, and that the fitness impacts of diets will be context‐dependent, including other foods that are included in the diet.

Cyanopeptolins are common secondary metabolites produced by cyanobacteria (Janssen, 2019). Among the proposed functions of these compounds is defense against parasites: during the process of infecting cyanobacteria, chytrid parasites engulf host cells and inject digestive proteases before extracting nutrients (Krarup et al., 1994). Cyanobacteria defend themselves by producing oligopeptides capable of inhibiting these digestive enzymes (Rohrlack et al., 2013). Cyanopeptolins also inhibit digestive proteases in other organisms, such as *Daphnia*, conferring a second function of protection against grazers. Here, we show that these compounds can also have negative impacts on parasites of *Daphnia*, demonstrating that these compounds can indirectly benefit herbivores that consume cyanobacteria by protecting them against infection. Protease inhibitors show pronounced seasonal fluctuations in natural phytoplankton (Kuster et al., 2013; Schwarzenberger et al., 2013); it would be interesting to see if these fluctuations are associated with the prevalence of *Metschnikowia* infections in *Daphnia*.

Understanding the function and mechanisms of secondary metabolites can allow us to discover and understand interactions occurring in the wild. This study benefited from the large literature about cyanobacteria oligopeptides, their function, and their impact on different model systems. As shown here, the molecular function need not change to have repercussions at different trophic levels. For freshwater ecosystems, this is particularly important because examples of co‐optation and self‐medication are lacking; while there are no descriptions of organism's self‐medication in aquatic systems to date, our results suggest self‐medication might be possible for herbivores that prey on cyanobacteria. Regardless of whether animals are able to self‐medicate, our results make it clear that defensive compounds produced by cyanobacteria can end up helping herbivores that consume them.

## AUTHOR CONTRIBUTIONS

**Kristel F. Sánchez:** Conceptualization (lead); data curation (lead); formal analysis (lead); funding acquisition (equal); investigation (lead); methodology (lead); project administration (lead); resources (equal); visualization (lead); writing – original draft (lead). **Eric von Elert:** Conceptualization (supporting); formal analysis (supporting); methodology (supporting); resources (supporting); writing – review and editing (supporting). **Kira Monell:** Investigation (supporting); methodology (supporting); writing – review and editing (supporting). **Siobhan Calhoun:** Investigation (supporting); methodology (supporting); writing – review and editing (supporting). **Aniqa Maisha:** Investigation (supporting); methodology (supporting); writing – review and editing (supporting). **Paige McCreadie:** Investigation (supporting); methodology (supporting); writing – review and editing (supporting). **Meghan A. Duffy:** Conceptualization (supporting); data curation (supporting); formal analysis (equal); funding acquisition (equal); investigation (equal); methodology (equal); project administration (supporting); resources (lead); supervision (supporting); visualization (equal); writing – original draft (supporting); writing – review and editing (lead).

## CONFLICT OF INTEREST STATEMENT

The authors declare no conflict of interest.

## Supporting information


Data S1.

## Data Availability

Data is accessible through DRYAD digital repository https://doi.org/10.5061/dryad.nzs7h44xq
