# Supplementary material for: Inhibition of gut digestive proteases by cyanobacterial diets decreases infection in a Daphnia host–parasite system
Source: Ecol Evol. 2024 Apr 19;14(4):e11340. doi: 10.1002/ece3.11340 (PMC11027009; doi:10.1002/ece3.11340)
Supplement: Supplementary file 1 — Data S1. [file ECE3-14-e11340-s001.docx]

**Supplementary materials**

**Enzyme activity assay methods**

On the day of the protease activity assay, we placed 100 µL of ice cold 0.1M potassium phosphate buffer (P-P buffer; pH 7.5) in each tube. We homogenized 20 *Daphnia* per tube in the buffer with a pestle and then centrifuged the tubes at 14,000 g for 3 min. We then transferred the supernatant into a new Eppendorf tube and stored on ice until use. In a single well of a 96-well plate, we mixed either 1.2 µL of 12.5 nM N-Succinyl-L-alanyl-L-alanyl-L-propyl-L-phenylalanine 4-nitroanilide (SucpNA; in DMSO) for chymotrypsin activity or 4.6 µL of 25 nM Benzoyl-arginine-p-nitrianilide (BapNA; in DMSO) with 128.8-147.8 µL of P-P buffer (depending on the *Daphnia* homogenate dose we used in the mixture, Table S4) and allowed the mixture to equilibrate for ~ 3 minutes. We then placed 1-20 µL (for a final well volume of 150 µL) of the *Daphnia* homogenate and immediately read the absorption at 390 nm. We read the absorption again at 15 min and then at 30 minutes to measure the increase in absorption at 390 nm. We repeated this for every clone ⅹ diet *Daphnia* homogenate. Replicates for each treatment varied depending on the amount of homogenate used, so the number of replicates varied from 2 to 5 replicates per clone ⅹ diet treatment.

We also measured the protein content of each *Daphnia* homogenate using the Coomassie Plus (Bradford) Assay Kit (Thermo Scientific) following the manufacturer’s instructions.

We calculated the activity as change of absorption per minute per µg of protein by subtracting the absorbance units (AU) at 390 nm at 30 minutes minus the AU at 390 nm at 0 minutes. We then divided this number by the minutes elapsed (30 minutes) and then divided by the total protein content added in the assay. Hence, we report proteolytic activity as ΔmAU/min/μg protein content.

**Statistical analyses**

We ran linear regressions between the proportion of infected individuals in a given cyanobacterial diet versus the proportion infected on the control diet *Scenedesmus;* this analysis corresponds to that presented in Figure 5 but compares other diets (replication information in Table S2)*.* We hypothesized that clones that had higher proportion infected on the control diet (as the ‘x’ variable) would have lower infection prevalence when feeding on a protective cyanobacterial diet (as the ‘y’ variable), yielding a slope, m, less than 1. A slope of 1 would mean there was no difference in susceptibility between the two diets.

To look at differences in within-host spore production we log-transformed the total number of spores found within an infected individual and ran an ANOVA with the log-transformed values as our response variables and diet, clone, and block as independent variables. We had 1-9 infected individuals per diet x clone treatment combination (mean: 3.2, median: 3; Table S2). We ran a Tukey’s post-hoc test to look at which diet treatments were different from each other.

We were interested in the effect of diet, clone, and infection status on reproduction (Table S2). Differences in host offspring production were analyzed using a GLMM with a gaussian family distribution. We ran two models, one with an interaction term of diet ⅹ infection status (yes or no infected) and one with no interaction term between those two terms. Offspring produced throughout the host lifetime was the response variable, while block and clone were treated as random effects.

**Results**

Diet and clone had an effect on the number of spores that were produced within a host (Table S3). *Microcystis aeruginosa* PCC 7806 WT significantly decreased the number of spores produced within a host (PCC WT μ=537600.6, PCC MT μ=668805.6, Scenedesmus μ=805916.7).

In both of our models, diet had an effect on the number of offspring produced by hosts throughout their lifetime (Figure S3; Table S3). Infection status and the interaction between infection status and diet did not have a significant effect on offspring production for our model that contains the interaction term (Table S3). In our second model with no interaction terms, we found an effect of infection status, which follows the pattern of infected animals having fewer offspring (Figure S3). The highest offspring reproduction was found for the *Scenedesmus* diet (mean offspring = 49.7) and, surprisingly, *Microcystis* CYA43 (mean offspring = 52.4). Since hosts in the cyanobacterial diet treatments were fed 50:50 green algae:cyanobacteria after parasite exposure, these results suggest that, for some diets, hosts may be able to compensate for gut protease inhibition, either by increasing feeding rates or producing different isozymes that allow them to still acquire enough amino acids for reproduction.


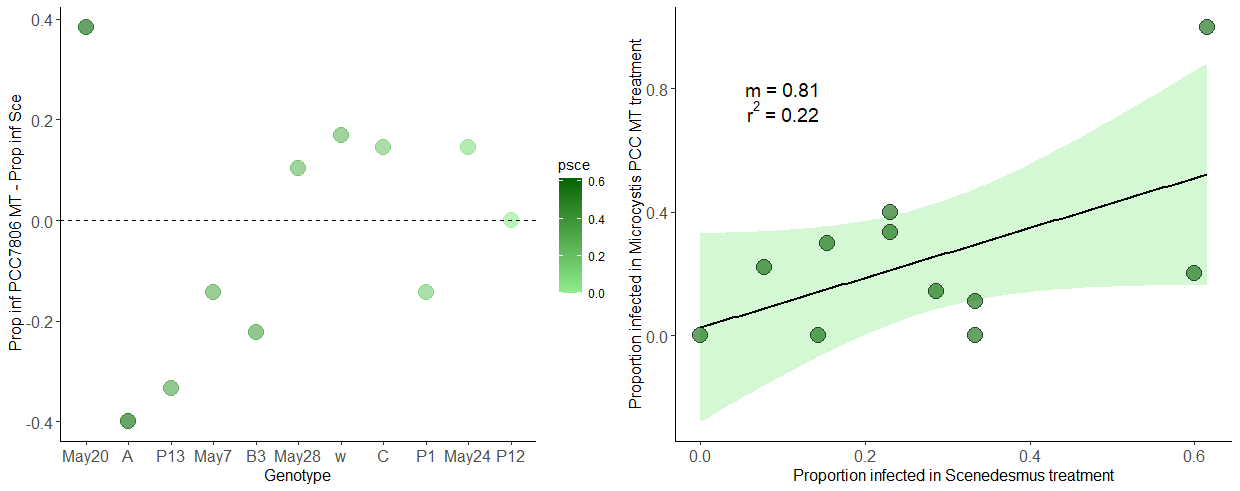

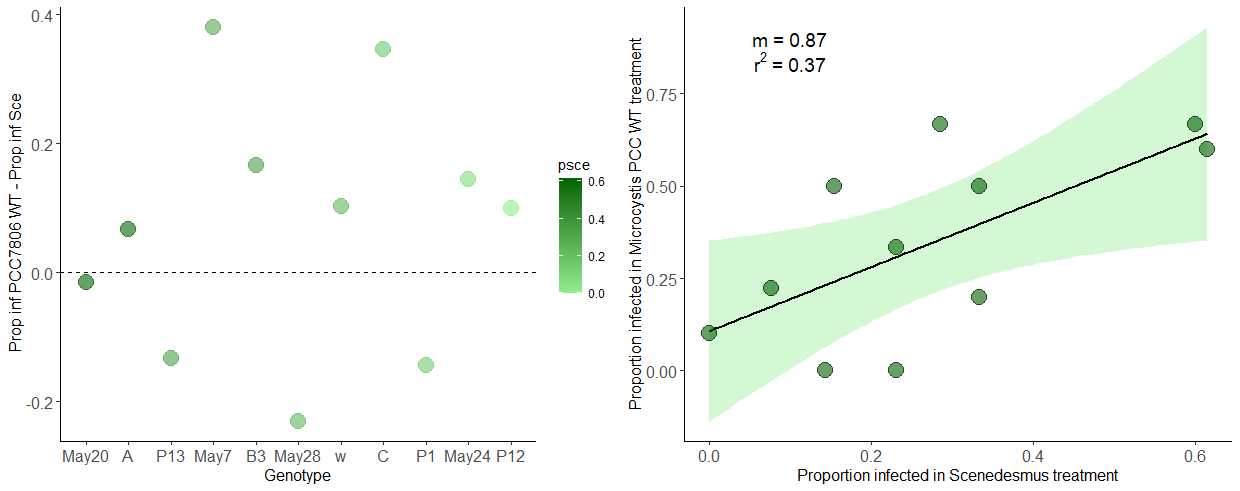

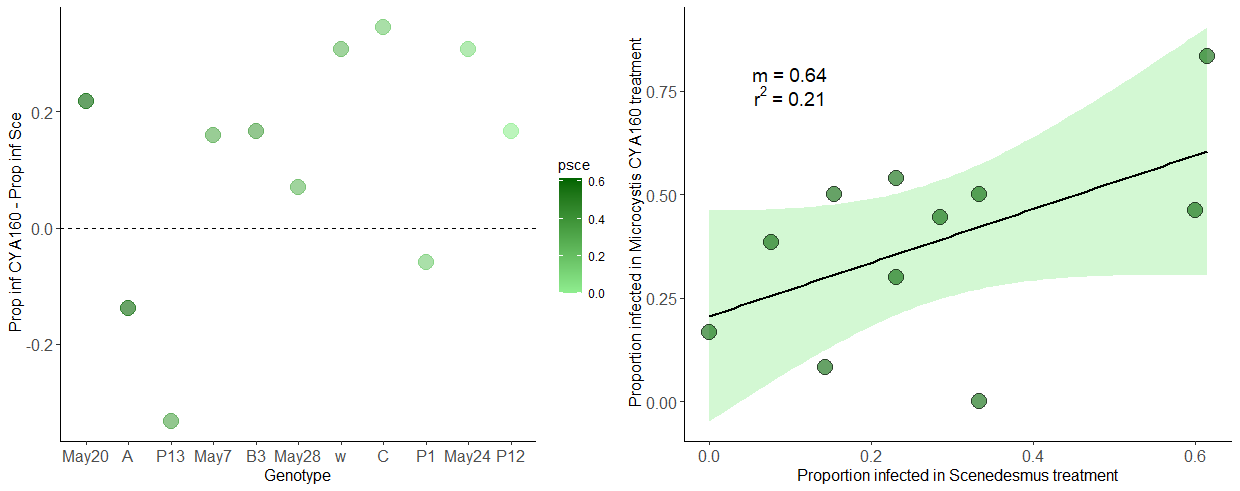


Figure S1. Effect size and linear regression of infection prevalence for Microcystis CYA160 (top panel), PCC7806 WT (middle panel), and PCC7806 MT (lower panel). Each point in both panels represents a clone.

**
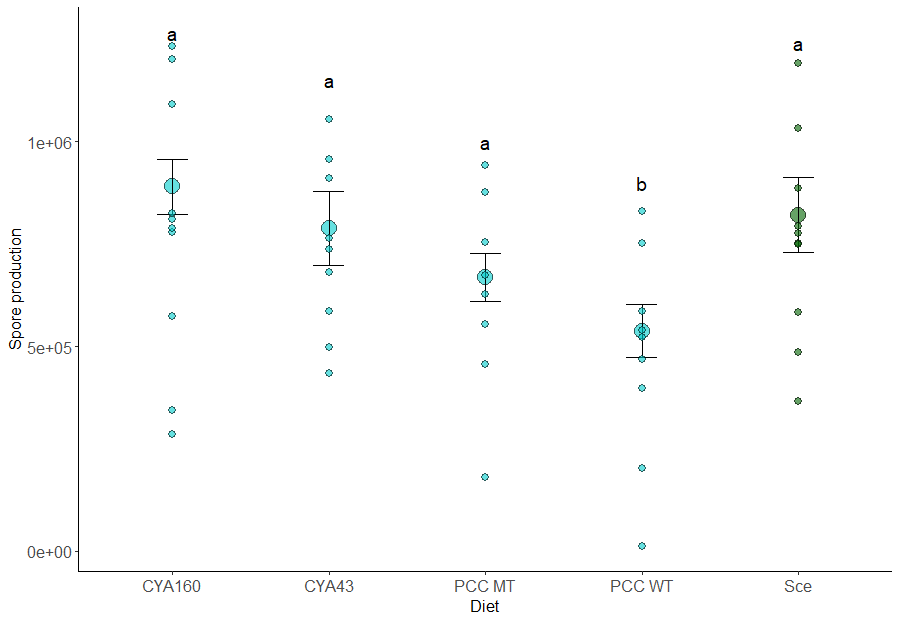
**

Figure S2. Within host parasite production. Large icons represent mean in treatments, while small icons represent per clone means within a given diet treatment. Error bars represent standard error. Letters represent Tukey’s post-hoc test results.


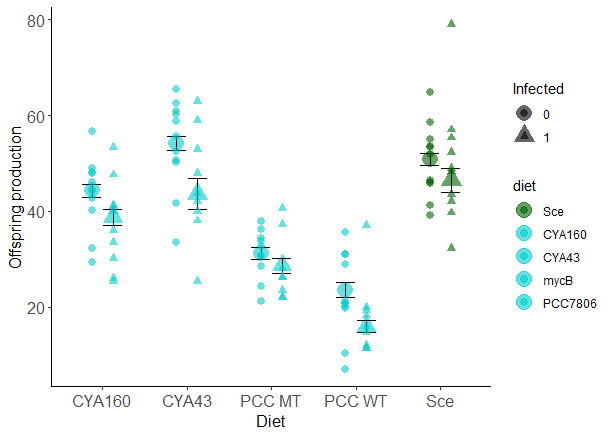


Figure S3. Host offspring production. Large icons are mean of treatment with standard errors. Small icons are mean offspring reproduction in a given clone line.

***Table S1*. Provenance of clones used in the study.** Please note that the citation list is not exhaustive for all the clones, the authors have only included papers relevant to this study.

| Clone | | Origin | Lake | Citations |
| --- | --- | --- | --- | --- |
| A | Sweden | | Bysjön | Schwarzenberger, A., Kuster, C. J., & Von Elert, E. (2012). Molecular mechanisms of tolerance to cyanobacterial protease inhibitors revealed by clonal differences in *Daphnia magna*. *Molecular Ecology*, *21*(19), 4898–4911. <https://doi.org/10.1111/j.1365-294X.2012.05753.x>  Schwarzenberger, A., Sadler, T., Motameny, S., Ben-khalifa, K., Frommolt, P., Altmüller, J., Konrad, K., & Von Elert, E. (2014). Deciphering the genetic basis of microcystin tolerance. *BMC Genomics*, *15*, 776. |
| B3 | Germany | | Binnensee | Schwarzenberger, A., Kuster, C. J., & Von Elert, E. (2012). Molecular mechanisms of tolerance to cyanobacterial protease inhibitors revealed by clonal differences in *Daphnia magna*. *Molecular Ecology*, *21*(19), 4898–4911. <https://doi.org/10.1111/j.1365-294X.2012.05753.x>  Schwarzenberger, A., Zitt, A., Kroth, P., Mueller, S., & Von Elert, E. (2010). Gene expression and activity of digestive proteases in *Daphnia*: effects of cyanobacterial protease inhibitors. *BMC Physiology*, *10*(6), 1-15. <https://doi.org/https://doi.org/10.1186/1472-6793-10-6>  Von Elert, E., Agrawal, M. K., Gebauer, C., Jaensch, H., & Bauer, U. (2004). Protease activity in gut of *Daphnia magn*a: evidence for trypsin and chymotrypsin enzymes. *Comparative Biochemistry and Physiology Part B*, *137*, 287–296. <https://doi.org/10.1016/j.cbpc.2003.11.008>  Von Elert, E., Zitt, A., & Schwarzenberger, A. (2012). Inducible tolerance to dietary protease inhibitors in *Daphnia magna*. *Journal of Experimental Biology*, *215*, 2051–2059. https://doi.org/10.1242/jeb.068742 |
| C | Sweden | | Bysjön | Schwarzenberger, A., Kuster, C. J., & Von Elert, E. (2012). Molecular mechanisms of tolerance to cyanobacterial protease inhibitors revealed by clonal differences in *Daphnia magna*. *Molecular Ecology*, *21*(19), 4898–4911. <https://doi.org/10.1111/j.1365-294X.2012.05753.x> |
| 20-May | Sweden | | Bysjön | Schwarzenberger, A., Ilić, M., & Von Elert, E. (2021). *Daphnia* populations are similar but not identical in tolerance to different protease inhibitors. *Harmful Algae*, *106,* 102062. https://doi.org/10.1016/j.hal.2021.102062 |
| 24-May | Sweden | | Bysjön | Schwarzenberger, A., Ilić, M., & Von Elert, E. (2021). *Daphnia* populations are similar but not identical in tolerance to different protease inhibitors. *Harmful Algae*, *106,* 102062. https://doi.org/10.1016/j.hal.2021.102062 |
| 28-May | Sweden | | Bysjön | Schwarzenberger, A., Ilić, M., & Von Elert, E. (2021). *Daphnia* populations are similar but not identical in tolerance to different protease inhibitors. *Harmful Algae*, *106,* 102062. https://doi.org/10.1016/j.hal.2021.102062 |
| 7-May | Sweden | | Bysjön | Schwarzenberger, A., Ilić, M., & Von Elert, E. (2021). *Daphnia* populations are similar but not identical in tolerance to different protease inhibitors. *Harmful Algae*, *106,* 102062. https://doi.org/10.1016/j.hal.2021.102062 |
| P1 | Poland | | Pond in Kampinoski National Park | Schwarzenberger, A., Ilić, M., & Von Elert, E. (2021). *Daphnia* populations are similar but not identical in tolerance to different protease inhibitors. *Harmful Algae*, *106,* 102062. https://doi.org/10.1016/j.hal.2021.102062 |
| P12 | Poland | | Pond in Kampinoski National Park | Schwarzenberger, A., Ilić, M., & Von Elert, E. (2021). *Daphnia* populations are similar but not identical in tolerance to different protease inhibitors. *Harmful Algae*, *106,* 102062. https://doi.org/10.1016/j.hal.2021.102062 |
| P13 | Poland | | Pond in Kampinoski National Park | Schwarzenberger, A., Ilić, M., & Von Elert, E. (2021). *Daphnia* populations are similar but not identical in tolerance to different protease inhibitors. *Harmful Algae*, *106,* 102062. https://doi.org/10.1016/j.hal.2021.102062 |
| W | Poland | | Pond near Warsaw | Schwarzenberger, A., Kuster, C. J., & Von Elert, E. (2012). Molecular mechanisms of tolerance to cyanobacterial protease inhibitors revealed by clonal differences in *Daphnia magna*. *Molecular Ecology*, *21*(19), 4898–4911. https://doi.org/10.1111/j.1365-294X.2012.05753.x |
| 17-May | Sweden | | Bysjön | Lange, J., Demir, F., Huesgen, P. F., Baumann, U., Von Elert, E. & Pichlo, C. (2018). Heterologous expression and characterization of a novel serine protease from *Daphnia magna*: A possible role in susceptibility to toxic cyanobacteria. *Aquatic Toxicology*, *205*, 140–147. https://doi.org/10.1016/j.aquatox.2018.09.013 |

***Table S2. Replication information.***

**Difference in infection on pairs of diets (Figure S1)**

| Scale of inference | Scale at which the factor of interest is applied | Number of replicates at the appropriate scale |
| --- | --- | --- |
| Comparison of clones fed different diets | Individual animals within a clone x diet treatment combination | 11 clones |

**Spore yield per infected host (Figure S2)**

| Scale of inference | Scale at which the factor of interest is applied | Number of replicates at the appropriate scale |
| --- | --- | --- |
| Comparison of clones and diets | Individual host (only hosts that became infected) | 1-9 per clone x diet combination (mean: 3.2, median: 3) |

**Reproduction per individual host (Figure S3)**

| Scale of inference | Scale at which the factor of interest is applied | Number of replicates at the appropriate scale |
| --- | --- | --- |
| Comparison of clones, diets, and infection status | Individual host | 1-13 individuals per clone x diet x infection status combination (mean: 5.7, median: 6.0) |

***Table S3*.** Statistical analyses results of data found in the supplementary material.

| Infection per diet linear regressions (all regression done against *Scenedesmus* infection proportion) | | | | |
| --- | --- | --- | --- | --- |
| InfΔ | **Estimate** | **Std. Error** | **t-value** | **p-value** |
| Mic CYA160 | 0.6469 | 0.3405 | 1.900 | 0.0899 |
|  | **r²** | 0.2069 | **F-statistic:** | 3.609 |
| Mic PCC WT | 0.8691 | 0.3283 | 2.648 | 0.0266 * |
|  | **r²** | 0.3754 | **F-statistic:** | 7.01 |
| Mic PCC MT | 0.80689 | 0.40824 | 1.976 | 0.0795 |
|  | **r²** | 0.2252 | **F-statistic:** | 3.907 |
| Spore production analyses | | | | |
| Anova | **Df** | **Sum Sq** | **F-value** | **P-value** |
| Diet | 4 | 9.03 | 2.879 | 0.02521 * |
| Clone | 10 | 19.84 | 2.529 | 0.00808 * |
| Block | 1 | 0.36 | 0.453 | 0.50201 |
| Offspring production GLMM | | | | |
| # of offspring ~ diet + Infection + (1\|Block) + (1\|clone) | | | | |
|  | **Df** | **Sum of Squares** | **F-value** | **p-value** |
| Diet | 4 | 63492 | 119.841 | < 2.2e-16 * |
| Infection | 1 | 1758 | 13.271 | 0.0002956 * |
| AIC | 4389.417 |  |  |  |

**Table S4. *Daphnia* homogenate (DH) dose used in proteases activity assay.** Volumes are given in µL. For cells that have more than one number, these are all the doses tried and used for analyses.

| ***Daphnia* genotype** | **Diet** | **DH volume of BApNA** | **P-P buffer volume** | **DH volume of SucpNA** | **P-P buffer volume** |
| --- | --- | --- | --- | --- | --- |
| A | *Scenedesmus* | 1 | 144.4 | 1 | 147.8 |
|  | *Microcystis* CYA43 | 1 | 144.4 | 1 | 147.8 |
|  | *Microcystis* CYA160 | 1.6 | 143.8 | 5 | 143.8 |
|  | *Microcystis* PCC WT | 2 | 143.4 | 1 | 147.8 |
|  | *Microcystis* PCC MT | 5 | 140.4 | 1 | 147.8 |
| B3 | *Scenedesmus* | 1.6 | 143.8 | 1 | 147.8 |
|  | *Microcystis* CYA43 | 1 | 144.4 | 5 & 10 | 143.8, 138.8 |
|  | *Microcystis* CYA160 | 1.6 | 143.8 | 1 | 147.8 |
|  | *Microcystis* PCC WT | 5 | 140.4 | 1 | 147.8 |
|  | *Microcystis* PCC MT | 5 | 140.4 | 2 | 146.8 |
| C | *Scenedesmus* | 1 | 144.4 | 1 | 147.8 |
|  | *Microcystis* CYA43 | 1 | 144.4 | 10 & 15 | 138.8 |
|  | *Microcystis* CYA160 | 2 | 143.4 | 1 | 147.8 |
|  | *Microcystis* PCC WT | 5 | 140.4 | 1 | 147.8 |
|  | *Microcystis* PCC MT | 5 & 10 | 140.4 & 135.4 | 1 | 147.8 |
| W | *Scenedesmus* | 1 | 144.4 | 0.6 | 148.2 |
|  | *Microcystis* CYA43 | 1 | 144.4 | 5 | 143.8 |
|  | *Microcystis* CYA160 | 1 | 144.4 | 0.6 | 148.2 |
|  | *Microcystis* PCC WT | 1 | 144.4 | 0.6 | 148.2 |
|  | *Microcystis* PCC MT | 1 | 144.4 | 0.6 | 148.2 |
| May7 | *Scenedesmus* | 2 | 143.4 | 1 | 147.8 |
|  | *Microcystis* CYA43 | 1 | 144.4 | 10 | 138.8 |
|  | *Microcystis* CYA160 | 2 | 143.4 | 1 | 147.8 |
|  | *Microcystis* PCC WT | 10 | 135.4 | 1 | 147.8 |
|  | *Microcystis* PCC MT | 10 | 135.4 | 1 | 147.8 |
| May20 | *Scenedesmus* | 1.6 | 143.8 | 0.6 | 148.2 |
|  | *Microcystis* CYA43 | 1.6 | 143.8 | 5 | 143.8 |
|  | *Microcystis* CYA160 | 1.6 | 143.8 | 0.6 | 148.2 |
|  | *Microcystis* PCC WT | 1.6 | 143.8 | 0.6 | 148.2 |
|  | *Microcystis* PCC MT | 1 | 144.4 | 0.6 | 148.2 |
| May24 | *Scenedesmus* | 1 | 144.4 | 1 | 147.8 |
|  | *Microcystis* CYA43 | 1 | 144.4 | 20 | 128.8 |
|  | *Microcystis* CYA160 | 1 | 144.4 | 1 | 147.8 |
|  | *Microcystis* PCC WT | 1.6 | 143.8 | 1 | 147.8 |
|  | *Microcystis* PCC MT | 5 | 140.4 | 1 | 147.8 |
| May28 | *Scenedesmus* | 1.6 | 143.8 | 1 | 147.8 |
|  | *Microcystis* CYA43 | 1 | 144.4 | 5 & 10 | 143.8, 138.8 |
|  | *Microcystis* CYA160 | 1.6 | 143.8 | 1 | 147.8 |
|  | *Microcystis* PCC WT | 2 | 143.4 | 1 | 147.8 |
|  | *Microcystis* PCC MT | 2 | 143.4 | 1 | 147.8 |
| P1 | *Scenedesmus* | 1 | 144.4 | 1 | 147.8 |
|  | *Microcystis* CYA43 | 2 | 143.4 | 10 | 138.8 |
|  | *Microcystis* CYA160 | 1 | 144.4 | 1 | 147.8 |
|  | *Microcystis* PCC WT | 2 | 143.4 | 1 | 147.8 |
|  | *Microcystis* PCC MT | 2 | 143.4 | 1 | 147.8 |
| P12 | *Scenedesmus* | 2 | 143.4 | 0.6 | 148.2 |
|  | *Microcystis* CYA43 | 1.6 | 143.8 | 5 | 143.8 |
|  | *Microcystis* CYA160 | 1.6 | 143.8 | 0.6 | 148.2 |
|  | *Microcystis* PCC WT | 2 | 143.4 | 0.6 | 148.2 |
|  | *Microcystis* PCC MT | 2 | 143.4 | 0.6 | 148.2 |
| P13 | *Scenedesmus* | 2 | 143.4 | 1 | 147.8 |
|  | *Microcystis* CYA43 | 2 | 143.4 | 1,2,5 & 10 | 147.8,  146.8, 143.8, 138.8 |
|  | *Microcystis* CYA160 | 1 | 144.4 | 1 | 147.8 |
|  | *Microcystis* PCC WT | 5 | 140.4 | 1 | 147.8 |
|  | *Microcystis* PCC MT | 5 | 140.4 | 1 | 147.8 |
